# Mass Mortality of Sea Lions Caused by Highly Pathogenic Avian Influenza A(H5N1) Virus

**DOI:** 10.3201/eid2912.230192

**Published:** 2023-12

**Authors:** Víctor Gamarra-Toledo, Pablo I. Plaza, Roberto Gutiérrez, Giancarlo Inga-Diaz, Patricia Saravia-Guevara, Oliver Pereyra-Meza, Elver Coronado-Flores, Antonio Calderón-Cerrón, Gonzalo Quiroz-Jiménez, Paola Martinez, Deyvis Huamán-Mendoza, José C. Nieto-Navarrete, Sandra Ventura, Sergio A. Lambertucci

**Affiliations:** Grupo de Investigaciones en Biología de la Conservación, Laboratorio Ecotono, INIBIOMA, Universidad Nacional del Comahue—CONICET, Bariloche, Argentina (V. Gamarra-Toledo, P.I. Plaza, S.A. Lambertucci);; Museo de Historia Natural, Universidad Nacional de San Agustín de Arequipa, Arequipa, Peru (V. Gamarra-Toledo, R. Gutiérrez);; Servicio Nacional de Áreas Naturales Protegidas por el Estado, Lima, Peru (R. Gutiérrez, G. Inga-Diaz, P. Saravia-Guevara, O. Pereyra-Meza, E. Coronado-Flores, A. Calderón-Cerrón, G. Quiroz-Jiménez, P. Martinez, D. Huamán-Mendoza, J.C. Nieto-Navarrete, S. Ventura);; Asociación Convive Perú, Madre de Dios, Perú (G. Inga-Diaz)

**Keywords:** influenza, viruses, avian influenza, HPAI, H5N1, sea lions, Peru, respiratory infections

## Abstract

We report a massive mortality of 5,224 sea lions (*Otaria flavescens*) in Peru that seemed to be associated with highly pathogenic avian influenza A(H5N1) virus infection. The transmission pathway may have been through the close contact of sea lions with infected wild birds. We recommend evaluating potential virus transmission among sea lions.

The panzootic (2020–2023) caused by the highly pathogenic avian influenza (HPAI) A(H5N1) caused numerous global outbreaks in 2022 (*1*). At the end of the year, the H5N1 virus reached South America, causing alarming bird mortalities in Peru (*2*). Comprehensive surveys suggest the virus killed >100,000 wild birds by the end of March 2023 only in protected areas (and >200,000 birds including other areas); particularly affected were Peruvian boobies (*Sula variegata*), guanay cormorants (*Leucocarbo bougainvilliorum*), and Peruvian pelicans (*Pelecanus thagus*) (*3*). The large biomass of infected wild birds may have led to a spillover event affecting marine mammals cohabiting with them, as reported in other parts of the world (*4*). Here, we report the death of several thousand sea lions (*Otaria flavescens*) on the coast of Peru within a few months; the sea lions manifested neurologic and respiratory signs. Clinical signs we observed suggest they were affected by HPAI H5N1, which was later confirmed by government and scientific reports (*5*,*6*).

During January–April 2023, we performed detailed surveillance of dead and agonal sea lions in protected marine areas of Peru (Figure). We found 5,224 animals dead or dying on beaches (Table). The synchronized high mortality rate we observed was concerning; up to 100 dead animals were found floating together in the sea, and 1,112 animals died on 1 island that has one of highest populations of sea lions in Peru (San Gallan, Ica, Reserva Nacional Paracas; Table). Those unprecedented massive mortalities for this region and even the entire world killed ≈5% of Peru’s population of this species in a few months (Figure, panels A, B; Appendix Figure) (*7*).

**Figure:**
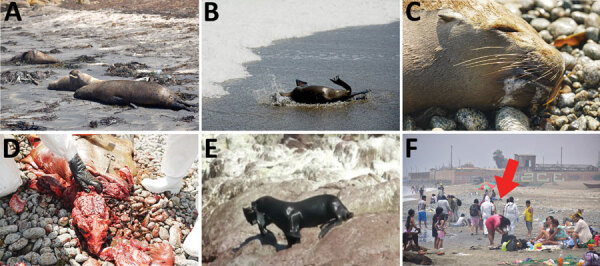
Sea lion deaths and investigation associated with outbreak of highly pathogenic avian influenza A(H5N1) in Paracas National Reserve, Peru, on the coastline, February 2023. A) Sea lion carcasses on the beach. B) Dying sea lion with ataxia. C) Dead sea lion with avian influenza clinical signs (whitish secretions). D) Sea lion necropsy showing a congestive brain. E) Sea lion trapping and eating a sick guanay cormorant, January 23, 2023. F) Field work sampling on a beach with a large number of bathers in the surroundings of infected carcasses. Red arrow indicates study staff wearing health protection equipment conducting field survey. Photograph credits: A, B, and D, Daniel Ampuero; C and F, Giancarlo Inga; E, Sandra Lizarme.

**Table:** Sea lion deaths potentially associated with highly pathogenic avian influenza virus A(H5N1) in protected areas of Peru, January–April 2023*

Date	Natural protected area	Island or guano island, if known	Clinical signs (no. animals)	Total deaths
January	RNPARACAS		Eyes closed and watering, nosebleeds and difficulty breathing (3); dyspnea, tremors (17)†	295
	RNSF		Carcasses floating in the sea (5)	25
	RNSIIPG	Chincha Sur	Found dead	46
		Punta San Juan	Convulsions (2)†	20
		Isla Cavinzas	Found dead	16
		Isla Asia	Carcasses floating in the sea (100)	240
		Isla Pachacamac	Found dead	7
		Isla Pescadores	Found dead	2
		Punta Lomitas	Foaming at the mouth, convulsions, paralysis of the forelimbs (1)	25
	ZRANCON		Found dead	9
	RNILLESCAS		Found dead	38
February	SNLM		Found dead	8
	RNPARACAS		Dyspnea, tremors (9)†	2,371
	RNSF		Found dead	691
	RNSIIPG	Chincha Norte	Found dead	16
		Chincha Sur	Found dead	10
		Punta San Juan	Found dead	325
		Punta Atico	Found dead	41
		Punta Coles	Found dead	104
		Isla Cavinzas	Found dead	3
		Isla Asia	Found dead	18
		Punta Lomas	Found dead	2
		Isla Palomino	Found dead	1
March	SNLM		Found dead	3
	RNPARACAS		Found dead	439
	RNSF		Found dead	82
	RNSIIPG	Chincha Norte	Found dead	2
		Chincha Sur	Found dead	8
		Punta San Juan	Found dead	6
		Punta Atico	Found dead	50
		Punta Coles	Found dead	108
		Isla Cavinzas	Found dead	1
		Isla Pachacamac	Found dead	1
		Isla Pescadores	Found dead	2
		Punta Lomas	Found dead	4
	ZRANCON		Found dead	4
April	SNLM		Found dead	3
	RNSIIPG	Isla Palomino	Found dead	2
		Chincha Norte	Found dead	7
		Punta San Juan	Found dead	89
	RNPARACAS		Found dead	91
	RNSF		Found dead	4
	RNILLESCAS		Found dead	5
Total				5,224

*Data for South American sea lions (*Otaria* *flavescens*). RNILLESCAS, Reserva Nacional Illescas; RNPARACAS, Reserva Nacional Paracas; RNSF, Reserva Nacional San Fernando; RNSIIPG, Reserva Nacional Sistema de Islas, Islotes y Puntas Guaneras; SNLM, Santuario Nacional Lagunas de Mejía; ZRANCÓN, Zona Reservada Ancón. †These animals died shortly after discovery.

National health authorities implemented restrictions regarding the manipulation of sick animals; for this reason, we were able to perform 1 necropsy, and the other observations were made by veterinarians at prudent distance. The clinical signs of agonal individuals were mainly neurologic, such as tremors, convulsions, and paralysis (Video 1; Video 2). The animals also showed respiratory signs such as dyspnea, tachypnea, and nasal and buccal secretions (Figure, panel C). The body condition of the necropsied sea lion ranged from good to very good. We observed substantial quantities of whitish secretions filling the upper respiratory tract (trachea and pharynx) (Figure, panel C). Lungs were congestive, with hemorrhagic focus compatible with interstitial pneumonia. Brain was also congestive, with hemorrhagic focus compatible with encephalitis (Figure, panel D). 

**Video 1:** Individual sea lion (*Otaria flavescens*) with acute respiratory distress and showing white foam from the mouth and nervous incoordination caused by highly pathogenic avian influenza A(H5N1) virus. The video was recorded on a beach in the city of Lima, Peru.

**Video 2:** Female sea lion (*Otaria flavescens*) with acute respiratory distress, aborting and exhibiting nervous incoordination consistent with highly pathogenic avian influenza A(H5N1) virus. The video was recorded on a beach near the city of Paracas, Peru.

Given the epidemiologic situation produced by HPAI H5N1 in wild birds that cohabit with the sea lions (*2*,*3*), the most plausible diagnosis causing this mass mortality event was acute disease caused by the virus. Clinical signs observed were similar to those reported in marine mammals infected with HPAI H5N1 in the United States (*4*). Official information from the Peru government and associated scientific research confirmed that not only birds but also sea lions tested positive for H5N1 virus (*3*,*5*,*6*). As of April 2023, sea lion deaths have surpassed 5,000 in Peru; thousands of sea lions with similar clinical signs died in Chile (*8*). This massive mortality event associated with HPAI H5N1 could be attributed to the large aggregations of sea lions that occur during the December–March breeding season (*9*).

In conclusion, sea lions in Peru experienced a deadly outbreak of disease that has caused mass deaths in several regions of the coastline (Figure). The sea lion mass mortality we described is compatible with systemic HPAI H5N1 that resulted in acute encephalitis and pneumonia. The source of the H5N1 virus affecting these sea lions was most probably the large number of infected live birds or their carcasses on the Peru coastline (*2*,*3*). Sea lions may be infected by close contact with those carcasses and through consuming them (Figure, panel E). The potential for direct transmission among sea lions from their colonial breeding behavior, in which they congregate by hundreds in the same area, should be evaluated, as should the large number of animals affected and the findings that many animals died simultaneously in groups in both Peru and Chile. Recent research described potential mammal-to-mammal infection in minks (*Neovison vison*) (*10*). In fact, unique mutations that merit further surveillance were found through viral sequencing of some of the deceased sea lions we surveyed (*5*).

Further research is required to confirm the HPAI H5N1 virus as the main factor affecting the sea lions and to address the transmission pathway in this social species. We call for more attention to human–infected animal interaction in this geographic region (Figure, panel F) to identify any rise in infections and prevent a new pandemic.

Appendix Additional information about mass mortality of marine mammals caused by highly pathogenic avian influenza A(H5N1) virus, Peru.
