# Supplementary figures and images for: Mass Mortality of Sea Lions Caused by Highly Pathogenic Avian Influenza A(H5N1) Virus
Source: Emerg Infect Dis. 2023 Dec;29(12):2553–6. doi: 10.3201/eid2912.230192 (PMC10683807; doi:10.3201/eid2912.230192)

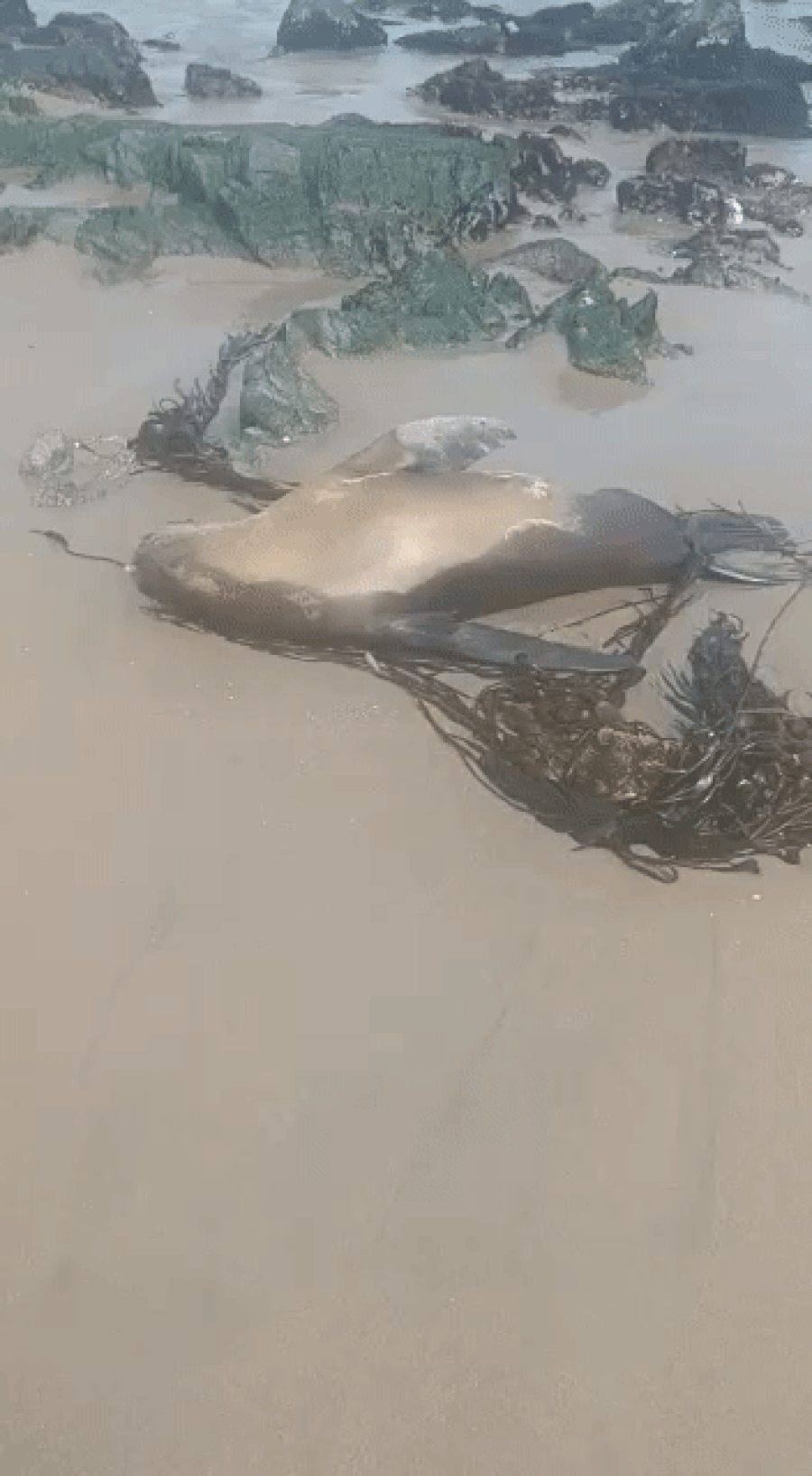

Supplement: Supplementary file 1 [file 23-0192-V1.gif]

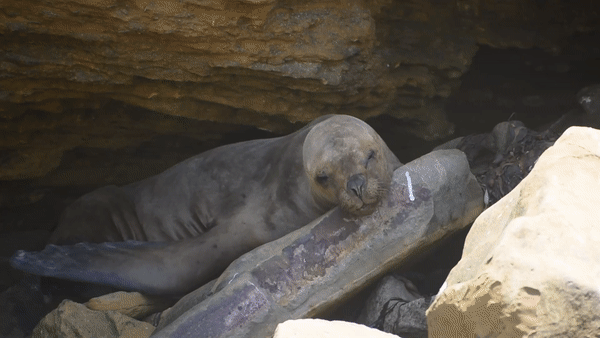

Supplement: Supplementary file 2 [file 23-0192-V2.gif]
