# Supplementary material for: Mass Mortality of Sea Lions Caused by Highly Pathogenic Avian Influenza A(H5N1) Virus
Source: Emerg Infect Dis. 2023 Dec;29(12):2553–6. doi: 10.3201/eid2912.230192 (PMC10683807; doi:10.3201/eid2912.230192)
Supplement: Appendix — Additional information about mass mortality of marine mammals caused by highly pathogenic avian influenza A(H5N1) virus, Peru. [file 23-0192-Techapp-s1.pdf]

Article DOI: http://doi.org/10.3201/eid2911.230192

EID cannot ensure accessibility for supplementary materials supplied by authors. Readers who have difficulty accessing supplementary content should contact the authors for assistance.

Mass Mortality of Sea Lions Caused by Highly Pathogenic Avian Influenza A(H5N1) Virus

Appendix

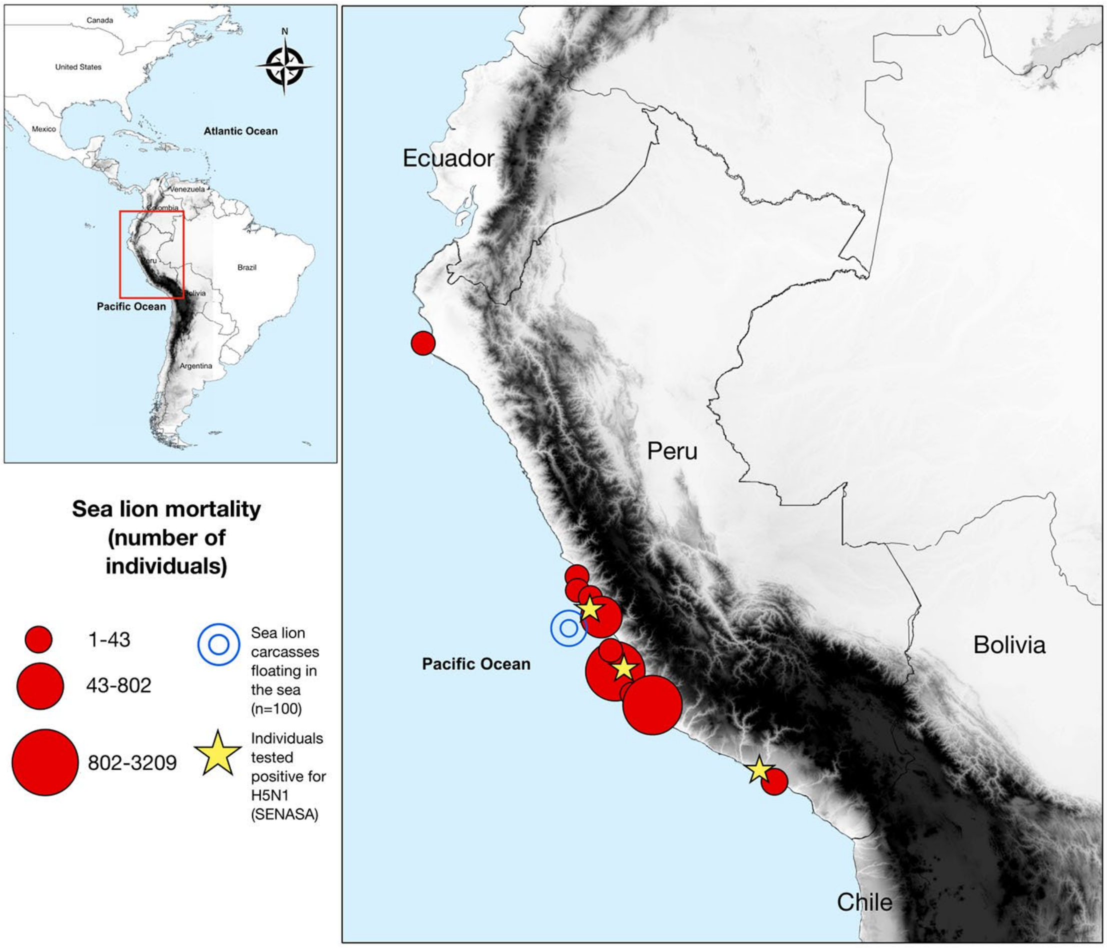

Appendix Figure. Geographic distribution of high mortalities in sea lions in January and April 2023 on the Peruvian coastline.
